# ERBB4 Perturbation Supports a Developmental Cytoskeletal Reactivation Hypothesis in Alzheimer’s Disease

**DOI:** 10.7759/cureus.117166

**Published:** 2026-09-29

**Authors:** Steven Lehrer

**Affiliations:** 1 Radiation Oncology, Icahn School of Medicine at Mount Sinai, New York, USA

**Keywords:** alzheimer’s disease, arp2/3 complex, cytoskeleton, erbb4, excitatory neurons

## Abstract

Background: Aberrant reactivation of developmental cytoskeletal programs may contribute to Alzheimer’s disease (AD). We tested whether a prespecified Arp2/3-RAC1-MYH10-KIF5C module is regulated by Erb-B2 receptor tyrosine kinase 4 (ERBB4) in an AD-model neuronal state, providing a perturbational test of the hypothesis that aberrant re-engagement of developmental cytoskeletal machinery contributes to AD.

Methods: We performed a secondary analysis of publicly available single-nucleus RNA-sequencing data from ERBB4 perturbation experiments using pseudobulk analysis with the primary conservative excitatory-neuron classifier. A prespecified 10-gene module comprising *Actr2, Actr3, Arpc1a, Arpc2, Arpc3, Arpc4, Arpc5, Rac1, Myh10,* and *Kif5c* was tested using CAMERA and expression-matched permutation analyses.

Results: ERBB4 disruption in 5 × FAD excitatory neurons downregulated all 10 module genes (CAMERA P = 0.0207). *Arpc4, Arpc1a,* and *Kif5c* remained significant after false-discovery-rate correction. Expression-matched permutation testing independently supported pathway downregulation (mean log-fold-change P = 0.0102; 10/10 genes down P = 0.0041; CAMERA permutation P = 0.0150). ERBB4 overexpression in wild-type neurons did not significantly alter the complete module, although *Arpc5* was significantly induced, suggesting selective component responsiveness.

Conclusions: Independent perturbational data support ERBB4-dependent regulation of a prespecified Arp2/3-centered cytoskeletal program in AD excitatory neurons, with effects dependent on the disease context.

## Introduction

Alzheimer’s disease (AD) is characterized by progressive synaptic dysfunction and loss, neuronal network abnormalities, reactive gliosis, and accumulation of amyloid-β and tau pathology [1]. Although amyloid and tau remain central features of AD, the cellular programs linking these pathological changes to synaptic failure and neurodegeneration are incompletely understood [2]. Increasing evidence suggests that AD may involve inappropriate engagement of developmental and structural programs that are normally tightly regulated in the mature brain [3]. Cytoskeletal remodeling is particularly relevant because maintenance of neuronal polarity, dendritic architecture, axonal transport, and synaptic stability depends on coordinated regulation of actin polymerization, actomyosin contractility, and microtubule-associated transport [4].

The Arp2/3 complex is a major regulator of branched actin nucleation and participates in neurite formation, dendritic spine remodeling, growth-cone dynamics, and cellular polarity [5]. *Rac1* (Rac family small GTPase 1) provides upstream regulation of actin remodeling [6], whereas non-muscle myosin IIB, encoded by *MYH10*, couples actin organization to contractile force. *KIF5C* contributes to microtubule-dependent neuronal transport [7]. We have proposed that inappropriate re-engagement or dysregulation of this interconnected machinery in mature neurons could contribute to AD by destabilizing neuronal architecture and synaptic function [3]. A critical unanswered question, however, is whether this cytoskeletal program responds experimentally to an upstream molecular driver of AD rather than simply representing a transcriptional correlate of established disease.

Lee et al. (2026) recently identified ectopic expression of Erb-B2 receptor tyrosine kinase 4 (ERBB4) in a population of early-responsive excitatory neurons as an early alteration in mouse models of AD. Single-nucleus RNA sequencing, together with gain- and loss-of-function experiments, implicated excitatory-neuronal ERBB4 in neuronal hyperactivity, abnormal glial synapse elimination, reactive gliosis, amyloid pathology, and cognitive dysfunction. Selective Erbb4 disruption in excitatory neurons of 5 × FAD mice ameliorated multiple disease-associated phenotypes, whereas ectopic Erbb4 expression in wild-type (WT) excitatory neurons reproduced several abnormalities [8]. These findings provide an experimentally tractable system for asking whether ERBB4-dependent AD pathology is accompanied by regulation of cytoskeletal machinery.

We therefore performed an independent secondary analysis of the publicly available ERBB4 perturbation data to test a cytoskeletal module defined previously in our developmental reactivation hypothesis of AD. Importantly, this gene set was specified before interrogation of the Lee et al. dataset [8] and was not derived from ERBB4-associated differential expression. Accordingly, we predicted that if ERBB4 contributes to aberrant engagement of this cytoskeletal program in the AD neuronal state, then Erbb4 disruption in 5 × FAD excitatory neurons should produce coordinated downregulation of the prespecified module.

The 10-gene module comprised seven Arp2/3 complex components (*Actr2, Actr3, Arpc1a, Arpc2, Arpc3, Arpc4, *and *Arpc5*), the polarity regulator *Rac1*, the actomyosin component *Myh10*, and the neuronal transport motor *Kif5c*. We tested whether this prespecified module was coordinately altered following Erbb4 disruption in 5 × FAD excitatory neurons and whether ectopic Erbb4 expression was sufficient to induce the corresponding program in WT neurons. By combining biological-replicate pseudobulk differential-expression analysis, competitive gene-set testing, and expression-matched permutation analysis, we sought to determine whether ERBB4 experimentally regulates an Arp2/3-centered cytoskeletal program in the AD neuronal state.

## Materials and methods

Data source and experimental design

Publicly available single-nucleus RNA-sequencing (snRNA-seq) data from the ERBB4 perturbation experiments reported by Lee et al. [7] were obtained from the NCBI Gene Expression Omnibus [8] (GEO; accession GSE292137). The analysis focused on the experiments in which Erbb4 was disrupted in 5 × FAD mice [9] and ectopically expressed in WT mice. The loss-of-function dataset comprised three sgControl-treated 5 × FAD biological replicates (GSM8850989-GSM8850991) and three sgErbb4-treated 5 × FAD replicates (GSM8850992-GSM8850994). The gain-of-function dataset comprised three HA control WT replicates (GSM8850995-GSM8850997) and three Erbb4-expressing WT replicates (GSM8850998-GSM8851000). These comparisons correspond to the ERBB4 perturbation experiments described by Lee et al.

The original Lee et al. cell-type and cluster annotations were not available as barcode-linked per-nucleus metadata in the GSE292137 GEO deposit; the deposited supplementary data consisted of 10x expression matrices, feature files, and barcode files. Excitatory nuclei were therefore identified independently from the deposited expression data using marker-based classification criteria.

Sensitivity analyses varied the minimum excitatory-neuron marker score from 0.05 to 0.15 and the required excess of the excitatory score over the inhibitory-neuron and maximum non-neuronal scores from 0.025 to 0.10. For each parameter combination, excitatory nuclei were reclassified from the raw count matrices, biological-replicate pseudobulk profiles were reconstructed, and the complete edgeR/CAMERA analysis was repeated using the same four-group design and prespecified contrast. Robustness was additionally quantified by barcode-level Jaccard similarity to the primary classification and genome-wide Pearson correlation of differential-expression log2 fold-change estimates.

Barcode-level agreement between the primary conservative and permissive excitatory-neuron classifications was high (mean Jaccard similarity = 0.959), indicating that the pathway result was not dependent on substantial changes in the nuclei included in the pseudobulk analysis.

Single-nucleus expression processing and excitatory-neuron identification

Gene-by-barcode count matrices, feature annotations, and barcode files were imported into R (R Foundation for Statistical Computing, Vienna, Austria) as sparse matrices. Gene identifiers were mapped to gene symbols using the supplied feature files. Individual nuclei were normalized to 10,000 total counts and log-transformed for cell-classification purposes. Excitatory neurons were identified using expression scores constructed from canonical excitatory-neuronal markers (*Slc17a7, Camk2a, Satb2, *and *Tbr1*) and exclusion scores representing inhibitory neurons (*Gad1, Gad2, *and *Slc6a1*), astrocytes (*Aqp4, Aldoc, Slc1a3, *and *Gfap*), microglia (*C1qa, C1qb, C1qc, *and *P2ry12*), oligodendrocytes (*Mbp, Plp1*, and *Mog*), oligodendrocyte precursor cells (*Pdgfra *and *Cspg4*), and endothelial cells (*Cldn5, Pecam1, *and *Kdr*). Nuclei were classified as excitatory when the excitatory-neuron score exceeded 0.10 and exceeded both the inhibitory-neuron and maximum non-neuronal scores by at least 0.05.

This conservative classification identified 3,513-6,356 excitatory nuclei per biological replicate, representing 33.8-52.4% of recovered nuclei. Sensitivity analysis using a less restrictive classification produced essentially identical pathway-level results.

Prespecified cytoskeletal gene set

The primary analysis used a 10-gene cytoskeletal module specified before examination of differential-expression results from the ERBB4 perturbation dataset. The module comprised seven components of the Arp2/3 branched-actin nucleation machinery (*Actr2, Actr3, Arpc1a, Arpc2, Arpc3, Arpc4, *and* Arpc5*), the polarity-associated small GTPase *Rac1*, the non-muscle myosin II component *Myh10*, and the neuronal microtubule motor *Kif5c*. The gene set was retained unchanged for all primary pathway and permutation analyses.

Pseudobulk differential-expression analysis

Raw counts from nuclei classified as excitatory neurons were summed within each biological replicate to generate sample-level pseudobulk count matrices. Biological samples, rather than individual nuclei, were treated as independent experimental units. Differential expression was analyzed in R using edgeR. Lowly expressed genes were removed using filterByExpr, library sizes were normalized using the trimmed mean of M-values method, and dispersions were estimated from the biological replicates. Quasi-likelihood generalized linear models were fitted using robust dispersion estimation. Prespecified contrasts compared sgErbb4 with sgControl in 5 × FAD mice and Erbb4 overexpression with HA control in WT mice. Gene-level P values were adjusted for multiple comparisons using the Benjamini-Hochberg false discovery rate (FDR).

Gene-set and permutation analyses

Coordinated regulation of the prespecified 10-gene module was evaluated using CAMERA, a competitive gene-set test that accounts for intergene correlation [10]. Loss- and gain-of-function contrasts were evaluated separately.

To assess whether the observed pathway effect could be explained by the expression characteristics of the selected genes, an independent matched permutation analysis was performed. Expressed genes were divided into 20 quantile bins according to baseline log counts per million, and 10,000 random 10-gene sets were generated by selecting genes from expression bins corresponding to those of the prespecified genes. Empirical one-sided P values were calculated for the mean log2 fold-change, median log2 fold-change, and number of genes showing downregulation. An additional 1,000 matched gene-set permutations were evaluated using CAMERA, and an empirical CAMERA P value was calculated as the proportion of matched random sets attaining a CAMERA P value equal to or smaller than that of the prespecified module. A fixed random seed was used to ensure reproducibility.

Simulation-based sensitivity analysis

Because each experimental condition contained three biological replicates, we performed a simulation-based sensitivity analysis using the expression-matched null distribution generated from 10,000 random 10-gene sets. Progressively larger coordinated mean log2 fold-change shifts were imposed on this empirical null distribution, and detection probability was defined as the proportion exceeding the one-sided 5% significance threshold for pathway downregulation. The analysis estimated the effect magnitude required for 80% detection probability and was intended as a sensitivity analysis rather than a retrospective power calculation based on the observed P value.

Statistical analysis

All analyses were performed in R 4.5 using the Matrix, data.table, and edgeR packages. Tests were two-sided for individual-gene differential-expression analyses unless otherwise specified; the matched permutation analyses tested the prespecified directional hypothesis of coordinated pathway downregulation following Erbb4 disruption. Statistical significance was defined as P < 0.05 for pathway-level analyses and FDR < 0.05 for genome-wide individual-gene analyses. Effect sizes are reported as log2 fold changes.

Data and code availability

The single-nucleus RNA-sequencing data analyzed in this study are publicly available from the NCBI Gene Expression Omnibus under accession GSE292137. The complete R code used for cell classification, pseudobulk construction, differential-expression analysis, CAMERA testing, expression-matched permutation analyses, sensitivity analyses, and figure generation is provided as Supplementary File 1.

## Results

ERBB4 disruption coordinately downregulates the prespecified cytoskeletal module

The ERBB4 perturbation dataset comprised three biological replicates, each of sgControl- and sgErbb4-treated 5 × FAD mice, and three replicates, each of HA control- and Erbb4-overexpressing wild-type (WT) mice. These samples were derived from hippocampal CA1 and correspond to the perturbation snRNA-seq experiments reported by Lee et al. [7]. Following conservative cell classification, 3,513-6,356 excitatory nuclei were retained per sample, representing 33.8-50.2% of recovered nuclei.

In 5 × FAD excitatory neurons, Erbb4 disruption produced a strikingly concordant effect on the prespecified 10-gene Arp2/3-RAC1-MYH10-KIF5C module. All 10 genes showed negative log2 fold-changes relative to sgControl. Competitive gene-set analysis confirmed significant coordinated downregulation of the module (CAMERA, P = 0.0207).

Among individual genes, the largest effect was observed for *Arpc4* (log2FC = -0.765, P = 3.54 × 10^-6^, FDR = 1.79 × 10^-4^). *Arpc1a* (log2FC = -0.273, P = 0.00684, FDR = 0.0287) and *Kif5c* (log2FC = -0.228, P = 0.00191, FDR = 0.0117) also remained significant after genome-wide FDR correction. *Actr2* was nominally decreased (log2FC = -0.131, P = 0.0417), while *Arpc2, Arpc3, Arpc5*, and *Rac1* showed concordant but individually non-significant reductions. In contrast, *Myh10* (log2FC = -0.012, P = 0.841) and Actr3 (log2FC = -0.002, P = 0.983) were essentially unchanged.

Excitatory-nucleus recovery by treatment group

Under the primary conservative classifier, the three sgControl 5 × FAD replicates contained 3,712/9,388 (39.5%), 3,513/10,407 (33.8%), and 4,519/9,599 (47.1%) excitatory nuclei, whereas the three sgErbb4 5 × FAD replicates contained 6,117/13,183 (46.4%), 6,263/14,097 (44.4%), and 6,356/12,657 (50.2%), respectively. The mean excitatory fraction was 40.1% in sgControl and 47.0% in sgErbb4 samples; this difference was not statistically significant at the biological-replicate level (Welch’s t-test, P = 0.209). Thus, excitatory-nucleus recovery was numerically higher following Erbb4 disruption, but the difference was not statistically significant across the three biological replicates per group.

Matched permutation analyses support pathway specificity

The coordinated decrease was not attributable to generalized transcriptional suppression following Erbb4 disruption. Across the transcriptome, 51.5% of analyzed genes were downregulated and 48.5% were upregulated, with a median log2FC of -0.013. In contrast, all 10 genes in the prespecified module were downregulated, with a mean log2FC of -0.219 and a median log2FC of -0.172.

Expression-matched permutation analysis using 10,000 random 10-gene sets demonstrated that the observed module was more strongly downregulated than expected from genes of comparable expression. The empirical P value was 0.0102 for mean log2FC and 0.0415 for median log2FC. Only 0.41% of matched random sets showed downregulation of all 10 genes (empirical P = 0.00410). An independent permutation analysis of the CAMERA statistic likewise supported the gene-set result (1,000 permutations; empirical P = 0.0150), consistent with the primary CAMERA P value of 0.0207.

ERBB4 overexpression does not induce the complete cytoskeletal module

The reciprocal gain-of-function experiment produced a different pattern. Ectopic Erbb4 expression in WT excitatory neurons did not significantly alter the prespecified 10-gene module as a whole (CAMERA P = 0.814). Thus, ERBB4 expression alone was insufficient to reproduce the coordinated pathway state observed in the 5 × FAD background.

Nevertheless, individual components showed selective responses to ERBB4 overexpression. Most notably, *Arpc5* increased significantly (log2FC = 0.548, P = 4.41 × 10^-4^, FDR = 0.0273), while *Rac1* increased nominally (log2FC = 0.218, P = 0.0282). The remaining genes did not show significant induction. These findings suggest that ERBB4-dependent regulation of the complete cytoskeletal module is context dependent, with coordinated pathway activity evident in the 5 × FAD disease state rather than being sufficient as a generalized consequence of ectopic ERBB4 expression.

Overall, the loss-of-function results provide independent perturbational evidence that ERBB4 regulates a prespecified Arp2/3-centered cytoskeletal program in AD-model excitatory neurons, while providing little evidence for specific regulation of Myh10. This distinction argues for an Arp2/3-centered rather than a complete Arp2/3-MYH10 mechanistic interpretation. Lee et al. independently demonstrated [7] that excitatory-neuronal ERBB4 disruption normalizes multiple AD-associated phenotypes, placing the observed cytoskeletal changes within an experimentally established disease-relevant perturbation.

Simulation-based sensitivity analysis indicated that, with three biological replicates per group and the observed expression-matched variability, a coordinated mean pathway effect of approximately -0.255 log2FC was required to achieve an estimated 80% detection probability. The observed mean effect of the prespecified module was -0.219 log2FC, indicating that the experiment was somewhat below the conventional 80% sensitivity benchmark for an effect of the observed magnitude.

Sensitivity to excitatory-neuron classification

The principal pathway result was highly robust to alternative excitatory-neuron definitions. Using the original permissive classifier, in which the excitatory marker score was required only to exceed inhibitory, astrocytic, microglial, and oligodendrocyte scores and be greater than zero, the prespecified module remained significantly downregulated after Erbb4 knockout (CAMERA P = 0.0197), with all 10 genes showing negative log2 fold changes. The primary conservative classifier produced an essentially identical result (CAMERA P = 0.0207; 10/10 genes down). Across additional threshold combinations, CAMERA P values ranged from 0.0207 to 0.0218, and genome-wide differential-expression estimates remained nearly identical to the primary analysis (Pearson r = 0.9986-1.000) (Table 1 and Figure 1). 

**Table 1:** Differential expression of the prespecified cytoskeletal module following Erbb4 disruption in 5 × FAD excitatory neurons. Gene-level edgeR pseudobulk differential expression estimates comparing sgErbb4 with sgControl in 5 × FAD excitatory neurons. CI: confidence interval; FDR: false discovery rate.

Gene	log2FC	95% CI	P value	FDR
Arpc4	-0.765	-1.089 to -0.441	3.54 × 10^-^⁶	1.79 × 10^-^⁴
Arpc2	-0.324	-0.659-0.012	0.0575	0.133
Arpc1a	-0.273	-0.490 to -0.056	0.00684	0.0287
Kif5c	-0.228	-0.372 to -0.083	0.00191	0.0117
Arpc3	-0.191	-0.520-0.138	0.256	0.394
Arpc5	-0.144	-0.391-0.103	0.25	0.387
Actr2	-0.131	-0.256 to -0.006	0.0417	0.105
Rac1	-0.097	-0.285-0.091	0.285	0.425
Myh10	-0.012	-0.137-0.112	0.841	0.897
Actr3	-0.002	-0.166-0.162	0.983	0.99

**Figure 1:**
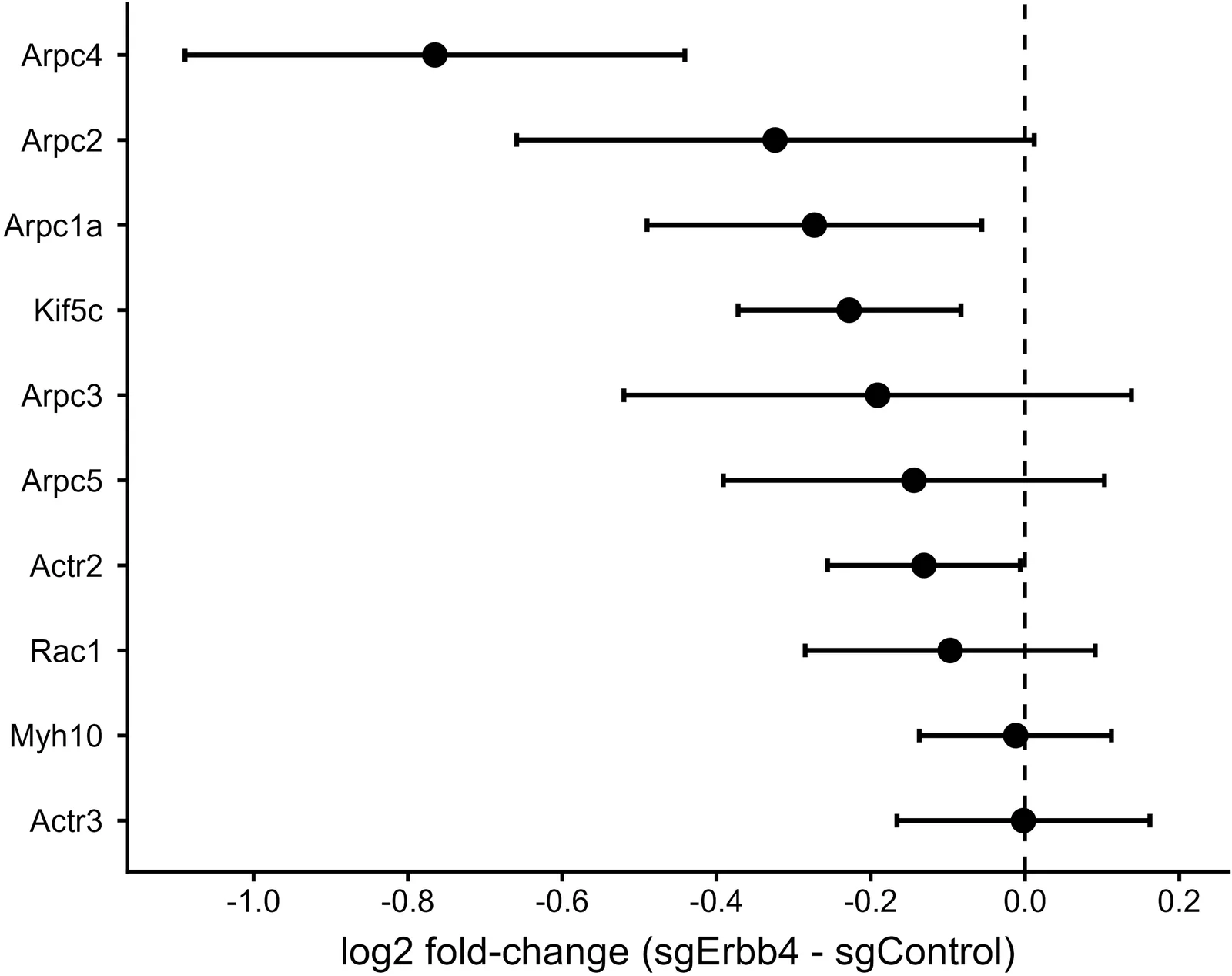
Erb-B2 receptor tyrosine kinase 4 (ERBB4) disruption downregulates a prespecified Arp2/3-centered cytoskeletal program in 5 × FAD excitatory neurons. Forest plot showing edgeR pseudobulk differential expression estimates for the prespecified 10-gene Arp2/3-RAC1-MYH10-KIF5C module in excitatory neurons following Erbb4 disruption in 5 × FAD mice (sgErbb4 vs. sgControl; three biological replicates per group). Points indicate log2 fold change, and horizontal bars indicate 95% confidence intervals. All 10 genes were downregulated, and the module showed significant coordinated downregulation by CAMERA (P = 0.0207). *Arpc4, Arpc1a*, and *Kif5c* remained significant after FDR correction (FDR < 0.05). Expression-matched permutation analyses independently supported pathway downregulation (mean log2 fold-change empirical P = 0.0102; 10 of 10 genes downregulated, P = 0.0041; CAMERA permutation P = 0.0150). ERBB4 overexpression in WT excitatory neurons did not significantly alter the complete module (CAMERA P = 0.814). Figure 1 was created by the author in R from the numerical differential-expression and confidence-interval estimates generated in this study; no generative artificial intelligence was used to create or modify the figure. WT: wild-type; FDR: false discovery rate; ERBB4: Erb-B2 receptor tyrosine kinase 4.

## Discussion

The asymmetry between loss- and gain-of-function experiments is informative. ERBB4 disruption in the 5 × FAD background coordinately suppressed the prespecified cytoskeletal module, whereas ERBB4 overexpression in WT neurons did not produce reciprocal pathway activation. This difference does not appear readily attributable to the absence of Erbb4 induction: strict excitatory-pseudobulk analysis using the primary conservative excitatory-neuron classifier demonstrated a 1.67-fold increase in Erbb4 expression in the overexpression condition (log2FC = 0.737, P = 0.0024), although the proportion of nuclei with detectable Erbb4 was unchanged. These findings suggest that increased ERBB4 expression alone is insufficient to activate the complete Arp2/3-centered program in WT excitatory neurons. ERBB4 regulation of this program may therefore be asymmetric or dependent on the disease-associated cellular context present in 5 × FAD neurons, including interacting signaling, transcriptional, or proteostatic factors. This interpretation remains provisional because transcript abundance does not directly establish the magnitude or distribution of ERBB4 protein overexpression.

The strongest individual response involved the Arp2/3 machinery. *Arpc4* was markedly reduced following Erbb4 disruption and remained significant after genome-wide multiple-testing correction, as did *Arpc1a*. *Actr2* was nominally reduced, while the remaining Arp2/3 components changed concordantly. These findings are notable because the Arp2/3 complex regulates branched actin nucleation underlying neuronal polarity, dendritic and synaptic architecture, and activity-dependent structural remodeling. *Kif5c*, a neuronal kinesin involved in microtubule-dependent transport, was also significantly reduced after Erbb4 disruption. Thus, the perturbational response extends beyond a single Arp2/3 subunit and is consistent with coordinated regulation of machinery responsible for neuronal structure and intracellular transport.

These observations provide a potential mechanistic extension of the findings of Lee et al. [7]. Their study identified ectopic ERBB4 expression in early-responsive excitatory neurons and demonstrated experimentally that excitatory-neuronal ERBB4 contributes to neuronal hyperactivity, abnormal synapse elimination, gliosis, amyloid pathology, and cognitive dysfunction. Our results suggest that an Arp2/3-centered cytoskeletal program may represent one component of the downstream cellular state associated with ERBB4 signaling. Importantly, the cytoskeletal module was specified before interrogation of the ERBB4 perturbation dataset, reducing the likelihood that the observed pathway association resulted from post-hoc selection of differentially expressed genes.

The loss- and gain-of-function experiments were not symmetrical. Whereas Erbb4 disruption significantly downregulated the complete module in the 5 × FAD background, ectopic Erbb4 expression in WT neurons did not significantly regulate the gene set as a whole (CAMERA P = 0.814). This result argues against a simple model in which ERBB4 alone is sufficient to activate the entire cytoskeletal program. Instead, engagement of the module may require the molecular environment of the AD brain, potentially involving amyloid-associated stress, altered neuronal activity, or other disease-state signals. This distinction between necessity in the disease state and sufficiency in otherwise normal neurons may be biologically informative.

To determine whether the absence of pathway-level activation in the overexpression experiment could be attributed to inadequate Erbb4 induction, we examined Erbb4 expression itself with pseudobulk analysis using the primary conservative excitatory-neuron classifier. Erbb4 expression was increased in the overexpression condition (log2FC = 0.737; fold-change = 1.67; P = 0.0024; FDR = 0.061). The proportion of strict excitatory nuclei with detectable Erbb4 was not increased (mean 6.53% in HA controls vs. 6.19% with Erbb4 overexpression; 0.95-fold; Wilcoxon P = 0.70), indicating that the pseudobulk increase reflected greater transcript abundance rather than a broad increase in the fraction of Erbb4-detectable nuclei. Despite this measurable increase in Erbb4 expression, the prespecified 10-gene cytoskeletal module was not coordinately activated (CAMERA P = 0.814).

The gain-of-function experiment nevertheless identified selective cytoskeletal responses. Most notably, *Arpc5* was significantly increased following ectopic Erbb4 expression in WT excitatory neurons (log2FC = 0.548, FDR = 0.0273), while Rac1 increased nominally. This suggests that particular components of branched-actin and polarity machinery may be especially responsive to ERBB4 signaling even when the complete cytoskeletal module is not engaged. The combination of decreased Arp2/3-related expression following ERBB4 loss in the AD state and selective induction following ERBB4 gain provides a potential link between the ectopic neuronal signaling identified by Lee et al. [7] and structural remodeling machinery.

The results also refine rather than fully confirm the proposed actomyosin model. *Myh10* showed little change following Erbb4 disruption and was not induced by ERBB4 overexpression. Consequently, these data provide substantially stronger evidence for an ERBB4-Arp2/3 cytoskeletal relationship than for ERBB4-dependent MYH10 contractility. The present findings therefore should not be interpreted as experimental validation of an entire Arp2/3-MYH10 mechanical oscillator. Rather, they provide independent perturbational evidence that an a priori Arp2/3-centered cytoskeletal module is regulated by ERBB4 in the AD neuronal state and identify specific components that merit direct mechanistic investigation.

The ERBB4 perturbation findings also provide an experimental complement to our previous observations in the human AD brain [3]. In the Mount Sinai Brain Bank single-nucleus data [11], the prespecified cytoskeletal machinery exhibited layer-specific associations with AD pathology, including increased MYH10 expression with tau tangle burden in Layer 4 and decreased ARPC2 expression in Layer 3, accompanied by increased KIF5C; amyloid burden was likewise associated with layer-dependent alterations in ARPC5 and MYH10 [3]. These human postmortem associations were necessarily observational and therefore could not determine whether the cytoskeletal program was downstream of a disease-relevant regulatory signal. The present ERBB4 perturbation analysis addresses that limitation from a complementary direction: disrupting Erbb4 in 5×FAD excitatory neurons shifted all 10 prespecified cytoskeletal genes downward, with significant pathway-level enrichment and expression-matched permutation support. Particularly notable is the convergence on Arp2/3 and axonal-transport components, because the original human analysis identified ARPC2 and KIF5C among the layer-specific signals, while the experimental dataset identifies *Arpc4*, *Arpc1a*, and *Kif5c* as the strongest FDR-significant responses. Thus, although the precise genes and directions differ across cell type, cortical layer, species, and experimental context, the two datasets independently implicate the same broader structural machinery. The perturbation results therefore strengthen the interpretation of the human associations by showing that an experimentally established AD-related signaling factor can regulate the prespecified cytoskeletal network rather than the network merely covarying with end-stage neuropathology.

A plausible signaling route linking ERBB4 to the Arp2/3 machinery is provided by the NRG1-ERBB4-PI3K-RAC1 axis [12]. NRG1-activated ERBB4 can engage PI3K signaling, while PI3K-generated PIP3 recruits Rac guanine-nucleotide exchange factors that activate RAC1; activated RAC1 in turn signals through the WAVE regulatory complex to stimulate Arp2/3-dependent branched actin assembly [13, 14]. Thus, ERBB4 could influence the prespecified cytoskeletal program through an established PI3K-RAC1-WAVE-Arp2/3 signaling architecture, although the present transcriptomic analysis does not establish which intermediates mediate the observed response.

The present findings were motivated by our previous hypothesis that AD may involve aberrant re-engagement of developmental cytoskeletal machinery that normally coordinates Arp2/3-RAC1-mediated branched-actin dynamics, MYH10-dependent actomyosin contractility, and KIF5C-dependent microtubule transport during neuronal polarization and morphogenesis [3]. In the original human postmortem analysis, components of this prespecified machinery showed cortical layer-specific associations with amyloid and tau pathology, but those cross-sectional observations could not determine whether the pathway was responsive to an upstream disease-associated signal. The ERBB4 perturbation experiment provides an independent and mechanistically distinct test of this idea: disruption of an experimentally established driver of AD-related excitatory-neuron dysfunction shifted all 10 prespecified genes in the same downward direction and significantly suppressed the module at the pathway level. Thus, the present findings strengthen the central premise that this developmental structural machinery is not merely associated with end-stage AD pathology but can be regulated downstream of a disease-relevant neuronal signaling perturbation. They do not, however, establish the complete proposed Arp2/3-MYH10 mechanical oscillator, because *Myh10* itself was essentially unchanged following ERBB4 perturbation.

Limitations

Several limitations should be considered. First, this was a secondary analysis of publicly available data from experiments designed to investigate ERBB4 rather than the cytoskeletal hypothesis tested here. Although the 10-gene module was prespecified before examination of differential-expression results, the present analysis was not part of the original experimental design.

Second, our analysis relied on computational identification of excitatory neurons because the original Lee et al. annotations were not publicly available and could not be obtained despite direct inquiry. Each experimental condition contained only three biological replicates, limiting statistical power and precluding detection of subtle effects. To assess the robustness of our cell-type classification, we repeated the full analysis pipeline across a range of excitatory-neuron score thresholds (0.05, 0.10, and 0.15). The principal CAMERA result was essentially unchanged (P = 0.0207-0.0218), genome-wide log₂FC values were nearly perfectly correlated across thresholds (Pearson r = 0.9986-1.000), and all 10 prespecified genes showed consistent downregulation under every classification examined. These sensitivity analyses suggest that our findings are not artifacts of a particular classification criterion, though independent replication with experimentally validated cell-type annotations remains desirable.

Third, excitatory neurons were identified computationally from canonical neuronal and non-neuronal marker expression rather than from the original authors' complete clustering annotations. However, the principal pathway result was essentially unchanged when a more stringent excitatory-neuron classification was applied (CAMERA P = 0.0197 vs. P = 0.0207), reducing concern that the finding was dependent on a particular classification threshold. Reproduction using the original cell annotations would nevertheless provide an important additional validation.

Fourth, transcript abundance does not establish corresponding changes in protein abundance, localization, actin polymerization, contractile force, or neuronal morphology [15]. The present data therefore demonstrate transcriptional regulation of cytoskeletal machinery rather than altered cytoskeletal mechanics themselves. Direct measurement of Arp2/3 proteins, actin architecture, neuronal polarity, and related structural phenotypes after ERBB4 perturbation will be required to establish the functional consequences of these transcriptional changes.

Finally, the full prespecified module was significantly affected only in the Erbb4 loss-of-function experiment. ERBB4 overexpression in WT neurons did not produce reciprocal pathway-level activation, and *Myh10* was essentially unchanged in both experiments. The findings therefore support disease-context-dependent regulation of an Arp2/3-centered cytoskeletal program but do not establish ERBB4 as sufficient to activate the complete proposed Arp2/3-MYH10 machinery.

## Conclusions

Independent ERBB4 perturbation data support ERBB4-dependent regulation of a prespecified Arp2/3-centered cytoskeletal program in the AD neuronal state, consistent with, but not demonstrating, aberrant engagement of developmental cytoskeletal machinery in AD. ERBB4 disruption in 5 × FAD excitatory neurons coordinately decreased all 10 components of a prespecified Arp2/3-RAC1-MYH10-KIF5C module, with significant pathway-level effects supported by expression-matched permutation testing. Because this module was defined before interrogation of the ERBB4 dataset, the findings provide an independent perturbational link between an established AD-related neuronal signaling factor and the developmental structural machinery implicated by our previous human postmortem observations. The strongest effects involved Arp2/3 and axonal-transport components, whereas MYH10 was essentially unchanged, supporting an ERBB4-Arp2/3-centered relationship rather than validation of the complete proposed mechanical oscillator. The absence of reciprocal pathway activation after ERBB4 overexpression in WT neurons further suggests that engagement of this machinery depends on the AD-associated cellular context.

## Appendices

# Supplementary File 1 # Complete R code for ERBB4 perturbation analysis (Cureus bracket-free edition) # GSE292137 # # IMPORTANT: # Cureus interprets ordinary R square-bracket indexing as numbered citations. # Therefore this Appendix version contains no ASCII square-bracket characters. # The helper slice() invokes R's ordinary extraction operator dynamically and # is analytically equivalent to standard R indexing. This formatting change # does not alter the analysis.

library(Matrix) library(data.table) library(edgeR) library(limma)

set.seed(12345)

# Dynamic ordinary R extraction without literal square brackets in source. slice <- function(x, ...) { extract_fun <- get(intToUtf8(91), envir = baseenv()) do.call(extract_fun, c(list(x), list(...))) }

cat("R version:", R.version.string, "\n") cat("Matrix:", as.character(packageVersion("Matrix")), "\n") cat("data.table:", as.character(packageVersion("data.table")), "\n") cat("edgeR:", as.character(packageVersion("edgeR")), "\n") cat("limma:", as.character(packageVersion("limma")), "\n") if (requireNamespace("BiocManager", quietly = TRUE)) { cat("Bioconductor:", as.character(BiocManager::version()), "\n") }

setwd("D:/alzsox9")

# ------------------------------------------------------------------------- # 1. Sample metadata # -------------------------------------------------------------------------

samples <- data.table( sample = c( "GSM8850989_sgc1", "GSM8850990_sgc2", "GSM8850991_sgc3", "GSM8850992_sge1", "GSM8850993_sge2", "GSM8850994_sge3", "GSM8850995_ha1", "GSM8850996_ha2", "GSM8850997_ha3", "GSM8850998_eb1", "GSM8850999_eb2", "GSM8851000_eb3" ), group = c( rep("sgControl_5xFAD", 3), rep("sgErbb4_5xFAD", 3), rep("HA_WT", 3), rep("Erbb4_WT", 3) ) )

module_genes <- c( "Actr2", "Actr3", "Arpc1a", "Arpc2", "Arpc3", "Arpc4", "Arpc5", "Rac1", "Myh10", "Kif5c" )

exc_markers   <- c("Slc17a7", "Camk2a", "Satb2", "Tbr1") inh_markers   <- c("Gad1", "Gad2", "Slc6a1") astro_markers <- c("Aqp4", "Aldoc", "Slc1a3", "Gfap") micro_markers <- c("C1qa", "C1qb", "C1qc", "P2ry12") oligo_markers <- c("Mbp", "Plp1", "Mog") opc_markers   <- c("Pdgfra", "Cspg4") endo_markers  <- c("Cldn5", "Pecam1", "Kdr")

# ------------------------------------------------------------------------- # 2. Read deposited 10x matrices # -------------------------------------------------------------------------

read_10x_sample <- function(prefix) { message("Reading ", prefix) mat <- Matrix::readMM(gzfile(paste0(prefix, "_matrix.mtx.gz"))) features <- fread(paste0(prefix, "_features.tsv.gz"), header = FALSE) barcodes <- fread(paste0(prefix, "_barcodes.tsv.gz"), header = FALSE) genes <- getElement(features, "V2") rownames(mat) <- make.unique(genes) colnames(mat) <- getElement(barcodes, "V1") as(mat, "dgCMatrix") }

counts_list <- setNames( lapply(samples$sample, function(s) read_10x_sample(file.path("geo", s))), samples$sample )

# ------------------------------------------------------------------------- # 3. Cell-classification scores # Each nucleus is normalized to 10,000 total counts and log1p transformed. # A marker score is the arithmetic mean across genes in that marker set. # Primary classifier: excitatory score > 0.10 and exceeds inhibitory and # maximum non-neuronal scores by at least 0.05. # -------------------------------------------------------------------------

marker_score <- function(logmat, genes) { genes <- intersect(genes, rownames(logmat)) if (length(genes) == 0) return(rep(0, ncol(logmat))) Matrix::colMeans(slice(logmat, genes, TRUE, drop = FALSE)) }

classify_excitatory <- function(counts, exc_cutoff = 0.10, margin = 0.05) { libsize <- Matrix::colSums(counts) keep <- libsize > 0 counts2 <- slice(counts, TRUE, keep, drop = FALSE) libsize2 <- slice(libsize, keep) norm <- t(t(counts2) / libsize2 * 10000) logmat <- log1p(norm)

  exc   <- marker_score(logmat, exc_markers) inh   <- marker_score(logmat, inh_markers) astro <- marker_score(logmat, astro_markers) micro <- marker_score(logmat, micro_markers) oligo <- marker_score(logmat, oligo_markers) opc   <- marker_score(logmat, opc_markers) endo  <- marker_score(logmat, endo_markers)

  nonneuronal_max <- pmax(astro, micro, oligo, opc, endo) excitatory <- exc > exc_cutoff & exc > inh + margin & exc > nonneuronal_max + margin names(excitatory) <- colnames(logmat)

  list( excitatory = excitatory, exc_score = exc, inh_score = inh, nonneuronal_score = nonneuronal_max ) }

# ------------------------------------------------------------------------- # 4. Primary conservative classification and pseudobulk construction # -------------------------------------------------------------------------

pb_list <- list() classification_stats_list <- list() primary_barcodes <- list()

for (s in samples$sample) { mat <- getElement(counts_list, s) cl <- classify_excitatory(mat, exc_cutoff = 0.10, margin = 0.05) exc_barcodes <- slice(names(cl$excitatory), cl$excitatory)

  primary_barcodes <- c(primary_barcodes, setNames(list(exc_barcodes), s)) classification_stats_list <- c( classification_stats_list, setNames(list(data.table( sample = s, n_total = ncol(mat), n_excitatory = length(exc_barcodes), pct_excitatory = 100 * length(exc_barcodes) / ncol(mat) )), s) )

  gene_counts <- Matrix::rowSums(slice(mat, TRUE, exc_barcodes, drop = FALSE)) pb_list <- c( pb_list, setNames(list(data.table(gene = rownames(mat), counts = as.numeric(gene_counts))), s) ) }

classification_stats <- rbindlist(classification_stats_list) print(classification_stats)

pb <- Reduce( function(x, y) merge(x, y, by = "gene", all = TRUE), lapply(seq_along(pb_list), function(i) { x <- copy(getElement(pb_list, i)) setnames(x, "counts", slice(samples$sample, i)) x }) )

for (nm in names(pb)) set(pb, which(is.na(getElement(pb, nm))), nm, 0) count_mat <- as.matrix(subset(pb, select = -gene)) rownames(count_mat) <- pb$gene count_mat <- slice(count_mat, TRUE, samples$sample, drop = FALSE)

# ------------------------------------------------------------------------- # 5. edgeR pseudobulk differential expression # -------------------------------------------------------------------------

group <- factor( samples$group, levels = c("sgControl_5xFAD", "sgErbb4_5xFAD", "HA_WT", "Erbb4_WT") ) design <- model.matrix(~0 + group) colnames(design) <- levels(group)

y <- DGEList(counts = count_mat) keep <- filterByExpr(y, design) y <- slice(y, keep, TRUE, keep.lib.sizes = FALSE) y <- calcNormFactors(y) y <- estimateDisp(y, design) fit <- glmQLFit(y, design, robust = TRUE)

contrast_KO <- makeContrasts( sgErbb4_5xFAD - sgControl_5xFAD, levels = design ) contrast_OE <- makeContrasts( Erbb4_WT - HA_WT, levels = design )

qlf_KO <- glmQLFTest(fit, contrast = contrast_KO) qlf_OE <- glmQLFTest(fit, contrast = contrast_OE) KO_table <- topTags(qlf_KO, n = Inf)$table OE_table <- topTags(qlf_OE, n = Inf)$table

KO_locked <- subset( data.table( gene = rownames(KO_table), logFC = KO_table$logFC, logCPM = KO_table$logCPM, F = KO_table$F, PValue = KO_table$PValue, FDR = KO_table$FDR ), gene %in% module_genes ) OE_locked <- subset( data.table( gene = rownames(OE_table), logFC = OE_table$logFC, logCPM = OE_table$logCPM, F = OE_table$F, PValue = OE_table$PValue, FDR = OE_table$FDR ), gene %in% module_genes )

print(slice(KO_locked, order(KO_locked$logFC), TRUE)) print(slice(OE_locked, order(OE_locked$logFC), TRUE))

# ------------------------------------------------------------------------- # 6. CAMERA competitive gene-set analysis # Same four-group design and prespecified KO/OE contrasts are used. # The default CAMERA inter-gene correlation handling is retained. # -------------------------------------------------------------------------

pathway_index <- which(rownames(y) %in% module_genes) camera_KO <- camera( y, index = list(Arp23_RAC1_MYH10_KIF5C = pathway_index), design = design, contrast = contrast_KO ) camera_OE <- camera( y, index = list(Arp23_RAC1_MYH10_KIF5C = pathway_index), design = design, contrast = contrast_OE ) print(camera_KO) print(camera_OE)

# ------------------------------------------------------------------------- # 7. Expression-matched permutation analysis # Matching abundance is the genome-wide edgeR KO logCPM measure. # Twenty quantile bins; 10,000 random 10-gene sets; seed 12345. # -------------------------------------------------------------------------

set.seed(12345) perm_dt <- data.table( gene = rownames(KO_table), logFC = KO_table$logFC, logCPM = KO_table$logCPM, PValue = KO_table$PValue ) perm_dt <- subset(perm_dt, is.finite(logFC) & is.finite(logCPM))

perm_dt$expr_bin <- cut( perm_dt$logCPM, breaks = unique(quantile( perm_dt$logCPM, probs = seq(0, 1, length.out = 21), na.rm = TRUE )), include.lowest = TRUE, labels = FALSE )

locked_dt <- subset( perm_dt, gene %in% module_genes, select = c(gene, logFC, logCPM, expr_bin) ) background <- subset(perm_dt, !gene %in% module_genes)

observed_mean_logFC <- mean(locked_dt$logFC) observed_median_logFC <- median(locked_dt$logFC) observed_n_down <- sum(locked_dt$logFC < 0)

n_perm <- 10000 perm_results <- data.table( permutation = seq_len(n_perm), mean_logFC = NA_real_, median_logFC = NA_real_, n_down = NA_integer_ )

for (b in seq_len(n_perm)) { random_genes <- character(0)

  for (j in seq_len(nrow(locked_dt))) { this_bin <- slice(locked_dt$expr_bin, j) pool_dt <- subset( background, expr_bin == this_bin & !gene %in% random_genes ) pool <- pool_dt$gene

    if (length(pool) == 0) { target_expr <- slice(locked_dt$logCPM, j) pool_dt <- subset(background, !gene %in% random_genes) ord <- order(abs(pool_dt$logCPM - target_expr)) ord <- head(ord, 100) pool <- slice(pool_dt$gene, ord) }

    random_genes <- c(random_genes, sample(pool, 1)) }

  x <- subset(background, gene %in% random_genes) set(perm_results, b, "mean_logFC", mean(x$logFC)) set(perm_results, b, "median_logFC", median(x$logFC)) set(perm_results, b, "n_down", sum(x$logFC < 0)) }

empirical_p_mean <- (1 + sum(perm_results$mean_logFC <= observed_mean_logFC)) / (n_perm + 1) empirical_p_median <- (1 + sum(perm_results$median_logFC <= observed_median_logFC)) / (n_perm + 1) empirical_p_all_down <- (1 + sum(perm_results$n_down >= observed_n_down)) / (n_perm + 1)

permutation_summary <- data.table( statistic = c("Mean logFC", "Median logFC", "Number genes down"), observed = c(observed_mean_logFC, observed_median_logFC, observed_n_down), empirical_P = c(empirical_p_mean, empirical_p_median, empirical_p_all_down) ) print(permutation_summary)

# ------------------------------------------------------------------------- # 8. CAMERA expression-matched permutation analysis: 1,000 sets # -------------------------------------------------------------------------

set.seed(12345) n_camera_perm <- 1000 camera_random_p <- numeric(n_camera_perm) locked_bins <- subset(locked_dt, gene %in% rownames(y)) background_camera <- subset( perm_dt, gene %in% rownames(y) & !gene %in% module_genes )

for (b in seq_len(n_camera_perm)) { random_genes <- character(0)

  for (j in seq_len(nrow(locked_bins))) { this_bin <- slice(locked_bins$expr_bin, j) pool_dt <- subset( background_camera, expr_bin == this_bin & !gene %in% random_genes ) pool <- pool_dt$gene

    if (length(pool) == 0) { target_expr <- slice(locked_bins$logCPM, j) pool_dt <- subset(background_camera, !gene %in% random_genes) ord <- head(order(abs(pool_dt$logCPM - target_expr)), 100) pool <- slice(pool_dt$gene, ord) }

    random_genes <- c(random_genes, sample(pool, 1)) }

  idx <- which(rownames(y) %in% random_genes) tmp <- camera( y, index = list(random = idx), design = design, contrast = contrast_KO ) camera_random_p <- replace(camera_random_p, b, head(tmp$PValue, 1)) }

observed_camera_p <- head(camera_KO$PValue, 1) empirical_camera_p <- (1 + sum(camera_random_p <= observed_camera_p)) / (n_camera_perm + 1) cat("CAMERA empirical P =", empirical_camera_p, "\n")

# ------------------------------------------------------------------------- # 9. Simulation-based sensitivity using the empirical matched-set null # Lower fifth percentile defines the one-sided detection threshold. # -------------------------------------------------------------------------

null_means <- perm_results$mean_logFC crit <- as.numeric(quantile(null_means, 0.05, na.rm = TRUE)) effect_grid <- c(0, -0.10, -0.20, -0.30, -0.40, -0.50)

power_results_fast <- data.table( log2FC = effect_grid, detection_probability = sapply( effect_grid, function(eff) mean(null_means + eff <= crit, na.rm = TRUE) ) ) print(power_results_fast)

observed_effect <- observed_mean_logFC observed_detection <- mean( null_means + observed_effect <= crit, na.rm = TRUE ) cat( "Detection probability at observed mean log2FC", observed_effect, "=", round(observed_detection, 3), "\n" )

fine_grid <- seq(0, -0.60, by = -0.005) fine_power <- sapply( fine_grid, function(eff) mean(null_means + eff <= crit, na.rm = TRUE) ) i80 <- head(which(fine_power >= 0.80), 1) cat( "Approximate log2FC for 80% detection:", slice(fine_grid, i80), "\nDetection probability:", slice(fine_power, i80), "\n" )

# ------------------------------------------------------------------------- # 10. Threshold-sweep robustness analysis # -------------------------------------------------------------------------

run_threshold <- function(counts_list, samples, exc_cutoff, margin) { pb_list2 <- list() stats_list2 <- list() barcode_sets <- list()

  for (s in samples$sample) { mat <- getElement(counts_list, s) cl <- classify_excitatory(mat, exc_cutoff = exc_cutoff, margin = margin) exc_barcodes <- slice(names(cl$excitatory), cl$excitatory)

    barcode_sets <- c(barcode_sets, setNames(list(exc_barcodes), s)) stats_list2 <- c( stats_list2, setNames(list(data.table( sample = s, n_total = ncol(mat), n_excitatory = length(exc_barcodes), pct_excitatory = 100 * length(exc_barcodes) / ncol(mat) )), s) )

    gene_counts <- Matrix::rowSums(slice(mat, TRUE, exc_barcodes, drop = FALSE)) pb_list2 <- c( pb_list2, setNames(list(data.table(gene = rownames(mat), counts = as.numeric(gene_counts))), s) ) }

  classification_stats2 <- rbindlist(stats_list2) pb2 <- Reduce( function(x, z) merge(x, z, by = "gene", all = TRUE), lapply(seq_along(pb_list2), function(i) { x <- copy(getElement(pb_list2, i)) setnames(x, "counts", slice(samples$sample, i)) x }) )

  for (nm in names(pb2)) set(pb2, which(is.na(getElement(pb2, nm))), nm, 0) count_mat2 <- as.matrix(subset(pb2, select = -gene)) rownames(count_mat2) <- pb2$gene count_mat2 <- slice(count_mat2, TRUE, samples$sample, drop = FALSE)

  group2 <- factor( samples$group, levels = c("sgControl_5xFAD", "sgErbb4_5xFAD", "HA_WT", "Erbb4_WT") ) design2 <- model.matrix(~0 + group2) colnames(design2) <- levels(group2)

  y2 <- DGEList(counts = count_mat2) keep2 <- filterByExpr(y2, design2) y2 <- slice(y2, keep2, TRUE, keep.lib.sizes = FALSE) y2 <- calcNormFactors(y2) y2 <- estimateDisp(y2, design2) fit2 <- glmQLFit(y2, design2, robust = TRUE)

  contrast_KO2 <- makeContrasts( sgErbb4_5xFAD - sgControl_5xFAD, levels = design2 ) qlf2 <- glmQLFTest(fit2, contrast = contrast_KO2) de2 <- topTags(qlf2, n = Inf)$table de2$gene <- rownames(de2)

  module_present <- intersect(module_genes, rownames(y2)) idx <- which(rownames(y2) %in% module_present) cam <- camera( y2, index = list(module = idx), design = design2, contrast = contrast_KO2 ) module_de <- subset(de2, gene %in% module_present) ko_samples <- subset( samples, group %in% c("sgControl_5xFAD", "sgErbb4_5xFAD") )$sample

  list( stats = data.table( exc_cutoff = exc_cutoff, margin = margin, camera_p = head(cam$PValue, 1), camera_direction = head(cam$Direction, 1), mean_module_logFC = mean(module_de$logFC, na.rm = TRUE), median_module_logFC = median(module_de$logFC, na.rm = TRUE), n_module_down = sum(module_de$logFC < 0, na.rm = TRUE), n_module_tested = nrow(module_de), mean_pct_excitatory = mean( subset(classification_stats2, sample %in% ko_samples)$pct_excitatory ) ), classification_stats = classification_stats2, barcode_sets = barcode_sets, de = de2, camera = cam ) }

threshold_grid <- CJ( exc_cutoff = c(0.05, 0.075, 0.10, 0.125, 0.15), margin = c(0.025, 0.05, 0.075, 0.10) )

threshold_results <- lapply(seq_len(nrow(threshold_grid)), function(i) { cat( "\nRunning", i, "of", nrow(threshold_grid), " cutoff =", slice(threshold_grid$exc_cutoff, i), " margin =", slice(threshold_grid$margin, i), "\n" ) run_threshold( counts_list = counts_list, samples = samples, exc_cutoff = slice(threshold_grid$exc_cutoff, i), margin = slice(threshold_grid$margin, i) ) })

sensitivity_table <- rbindlist(lapply(threshold_results, function(x) x$stats)) baseline_idx <- which( threshold_grid$exc_cutoff == 0.10 & threshold_grid$margin == 0.05 ) baseline_result <- getElement(threshold_results, baseline_idx) baseline_barcodes <- baseline_result$barcode_sets

jaccard_results <- rbindlist(lapply(seq_along(threshold_results), function(i) { current_result <- getElement(threshold_results, i) vals <- sapply(samples$sample, function(s) { a <- getElement(baseline_barcodes, s) b <- getElement(current_result$barcode_sets, s) length(intersect(a, b)) / length(union(a, b)) }) data.table( exc_cutoff = slice(threshold_grid$exc_cutoff, i), margin = slice(threshold_grid$margin, i), mean_jaccard = mean(vals), min_jaccard = min(vals) ) }))

sensitivity_table <- merge( sensitivity_table, jaccard_results, by = c("exc_cutoff", "margin") )

baseline_de <- subset(baseline_result$de, select = c(gene, logFC)) setnames(baseline_de, "logFC", "baseline_logFC")

cor_results <- rbindlist(lapply(seq_along(threshold_results), function(i) { current_result <- getElement(threshold_results, i) this_de <- subset(current_result$de, select = c(gene, logFC)) setnames(this_de, "logFC", "threshold_logFC") z <- merge(baseline_de, this_de, by = "gene") data.table( exc_cutoff = slice(threshold_grid$exc_cutoff, i), margin = slice(threshold_grid$margin, i), genomewide_r = cor( z$baseline_logFC, z$threshold_logFC, use = "complete.obs" ) ) }))

sensitivity_table <- merge( sensitivity_table, cor_results, by = c("exc_cutoff", "margin") ) setorder(sensitivity_table, exc_cutoff, margin) print(sensitivity_table) fwrite(sensitivity_table, "ERBB4_excitatory_threshold_sensitivity.csv")

# ------------------------------------------------------------------------- # 11. Original permissive-classifier endpoint # -------------------------------------------------------------------------

normalize_counts <- function(mat) { lib <- Matrix::colSums(mat) lib <- replace(lib, lib == 0, 1) norm <- t(t(mat) / lib * 10000) norm@x <- log1p(norm@x) norm }

classify_excitatory_permissive <- function(mat) { norm <- normalize_counts(mat) ex  <- marker_score(norm, exc_markers) inh <- marker_score(norm, inh_markers) ast <- marker_score(norm, astro_markers) mic <- marker_score(norm, micro_markers) oli <- marker_score(norm, oligo_markers) keep <- ex > inh & ex > ast & ex > mic & ex > oli & ex > 0

  data.table( barcode = colnames(mat), ex_score = ex, inh_score = inh, astro_score = ast, micro_score = mic, oligo_score = oli, excitatory = keep ) }

run_permissive_endpoint <- function(counts_list, samples) { pb_list3 <- list() stats_list3 <- list() barcode_sets <- list()

  for (s in samples$sample) { mat <- getElement(counts_list, s) cls <- classify_excitatory_permissive(mat) exc_barcodes <- subset(cls, excitatory)$barcode

    barcode_sets <- c(barcode_sets, setNames(list(exc_barcodes), s)) stats_list3 <- c( stats_list3, setNames(list(data.table( sample = s, n_total = ncol(mat), n_excitatory = length(exc_barcodes), pct_excitatory = 100 * length(exc_barcodes) / ncol(mat) )), s) )

    gene_counts <- Matrix::rowSums(slice(mat, TRUE, exc_barcodes, drop = FALSE)) pb_list3 <- c( pb_list3, setNames(list(data.table(gene = rownames(mat), counts = as.numeric(gene_counts))), s) ) }

  classification_stats3 <- rbindlist(stats_list3) pb3 <- Reduce( function(x, z) merge(x, z, by = "gene", all = TRUE), lapply(seq_along(pb_list3), function(i) { x <- copy(getElement(pb_list3, i)) setnames(x, "counts", slice(samples$sample, i)) x }) )

  for (nm in names(pb3)) set(pb3, which(is.na(getElement(pb3, nm))), nm, 0) count_mat3 <- as.matrix(subset(pb3, select = -gene)) rownames(count_mat3) <- pb3$gene count_mat3 <- slice(count_mat3, TRUE, samples$sample, drop = FALSE)

  group3 <- factor( samples$group, levels = c("sgControl_5xFAD", "sgErbb4_5xFAD", "HA_WT", "Erbb4_WT") ) design3 <- model.matrix(~0 + group3) colnames(design3) <- levels(group3)

  y3 <- DGEList(counts = count_mat3) keep3 <- filterByExpr(y3, design3) y3 <- slice(y3, keep3, TRUE, keep.lib.sizes = FALSE) y3 <- calcNormFactors(y3) y3 <- estimateDisp(y3, design3) fit3 <- glmQLFit(y3, design3, robust = TRUE)

  contrast_KO3 <- makeContrasts( sgErbb4_5xFAD - sgControl_5xFAD, levels = design3 ) qlf3 <- glmQLFTest(fit3, contrast = contrast_KO3) de3 <- topTags(qlf3, n = Inf)$table de3$gene <- rownames(de3)

  module_present <- intersect(module_genes, rownames(y3)) idx <- which(rownames(y3) %in% module_present) cam <- camera( y3, index = list(module = idx), design = design3, contrast = contrast_KO3 ) module_de <- subset(de3, gene %in% module_present)

  list( stats = data.table( classifier = "Permissive", camera_p = head(cam$PValue, 1), camera_direction = head(cam$Direction, 1), mean_module_logFC = mean(module_de$logFC, na.rm = TRUE), median_module_logFC = median(module_de$logFC, na.rm = TRUE), n_module_down = sum(module_de$logFC < 0, na.rm = TRUE), n_module_tested = nrow(module_de) ), classification_stats = classification_stats3, barcode_sets = barcode_sets, de = de3, camera = cam ) }

permissive_test <- run_permissive_endpoint(counts_list, samples) perm_jaccard <- sapply(samples$sample, function(s) { a <- getElement(baseline_barcodes, s) b <- getElement(permissive_test$barcode_sets, s) length(intersect(a, b)) / length(union(a, b)) })

cat( "Mean Jaccard, permissive vs primary =", mean(perm_jaccard), "\nMinimum Jaccard =", min(perm_jaccard), "\n" )

strict_de <- subset(baseline_result$de, select = c(gene, logFC)) setnames(strict_de, "logFC", "strict_logFC") perm_de <- subset(permissive_test$de, select = c(gene, logFC)) setnames(perm_de, "logFC", "permissive_logFC") z <- merge(strict_de, perm_de, by = "gene")

cat( "Genome-wide logFC correlation, permissive vs primary =", cor(z$strict_logFC, z$permissive_logFC, use = "complete.obs"), "\n" ) print(permissive_test$stats) print(permissive_test$camera)

# ------------------------------------------------------------------------- # 12. Erbb4 overexpression check using the primary conservative classifier # -------------------------------------------------------------------------

erbb4_rows <- grep("^Erbb4$", rownames(OE_table), ignore.case = TRUE) erbb4_result <- slice(OE_table, erbb4_rows, TRUE, drop = FALSE) cat( "\nErbb4 log2FC =", erbb4_result$logFC, "\nFold change =", 2^erbb4_result$logFC, "\nP =", erbb4_result$PValue, "\nFDR =", erbb4_result$FDR, "\n" )

# ------------------------------------------------------------------------- # 13. Principal outputs and session information # -------------------------------------------------------------------------

fwrite(classification_stats, "ERBB4_primary_classification_stats.csv") fwrite(KO_locked, "ERBB4_KO_prespecified_module.csv") fwrite(OE_locked, "ERBB4_OE_prespecified_module.csv") fwrite(permutation_summary, "ERBB4_expression_matched_permutation_summary.csv")

cat("\nSession information\n") print(sessionInfo())
 
